# Dental Manifestations and Celiac Disease—An Overview

**DOI:** 10.3390/jcm12082801

**Published:** 2023-04-10

**Authors:** Herbert Wieser, Massimo Amato, Mario Caggiano, Carolina Ciacci

**Affiliations:** 1 Hamburg School of Food Science, Institute of Food Chemistry, University of Hamburg, 20146 Hamburg, Germany; h.wieser2@gmx.de; 2 Department of Medicine, Surgery, Dentistry Scuola Medica Salernitana, University of Salerno, 84081 Baronissi, Italy

**Keywords:** caries, celiac disease, dental age, dental enamel defects, dental plaque, periodontitis

## Abstract

This review summarizes recent investigations on dental manifestations in celiac disease. Particular attention is paid to delayed dental eruption and maturity, dental enamel defects, molar incisor hypomineralization, dental caries, dental plaque, and periodontitis. Most studies confirmed a higher frequency of delayed dental eruption and maturation in children and dental enamel defects in children and adults with celiac disease compared to healthy individuals. The malabsorption of various micronutrients, especially calcium and vitamin D, as well as immunity, is considered the main cause of these conditions. An early diagnosis of celiac disease and introducing a gluten-free diet might prevent the development of these conditions. Otherwise, the damage has already been established, and it is irreversible. Dentists can play an important role in identifying people who may have unrecognized celiac disease and may help prevent its progress and long-term complications. Investigations on dental caries, plaque, and periodontitis in celiac disease are rare and inconsistent; these complaints need further examination.

## 1. Introduction

Celiac disease (CeD) is a chronic immune-mediated small-intestinal enteropathy precipitated by the exposure of genetically predisposed individuals to dietary gluten proteins (recently reviewed by Caio et al., 2019 [1]; Lindfors et al., 2019 [2]; Oxentenko and Rubio-Tapia, 2019 [3]). The global prevalence of CeD has been estimated at around 1.7% based on positive serology and 0.7% based on biopsy-confirmed CeD [4]. The genetic predisposition includes the human leukocyte antigen (HLA) class II alleles HLA-DQ2.5, -DQ2.2, -DQ7, and -DQ8 and a number of non-HLA genes. Environmental factors such as infections, imbalanced small-intestinal microbiota, and increased intestinal permeability have additionally been associated with the development of CeD. The pathomechanism of CeD consists of the incomplete digestion of gluten proteins in the gastrointestinal tract, resulting in the release of immunogenic peptides, the para- and transcellular passage of these peptides through the intestinal epithelium, and the combined adaptive and innate immune responses to the peptides in the lamina propria. Pathologically, CeD is characterized by damage to the small-intestinal mucosa (“flattened mucosa”) including villous atrophy, crypt hyperplasia, and increased lymphocyte infiltration of the epithelium. The diagnostic scheme of CeD is based on symptoms typical of CeD, the testing of serum antibodies, and a histological judgement of duodenal biopsies. Lifelong strict adherence to a gluten-free diet (GFD) is currently the only effective treatment of CeD, and its benefits for most patients are obvious.

Patients with CeD may present with various signs and symptoms or even no symptoms at all [5]: the presentation of symptomatic CeD is extremely variable and consists of intestinal and/or extra-intestinal symptoms [6]. Classical intestinal symptoms are chronic diarrhea, bloating, and abdominal pain, for instance. Extra-intestinal manifestations include conditions caused by deficiencies of essential nutrients (e.g., anemia and osteoporosis), reproductive problems (e.g., pregnancy complications and infertility), neurological disorders (e.g., epilepsy and migraine), and psychiatric complaints (e.g., depression and schizophrenia). 

Numerous studies on the associations between CeD and extra-intestinal symptoms underlined that certain dental manifestations are well-recognized signs of CeD. In patients with as-yet-undiagnosed CeD, these can sometimes be the only presenting features. Typical manifestations, majorly related to pediatric CeD, include delayed dental eruption and maturity, dental enamel defects (DEDs), molar incisor hypomineralization, dental caries, dental plaque, and periodontitis (reviewed by Krzywicka et al., 2014 [7]; Macho et al., 2017 [8]; van Gils et al., 2017 [9]). Dentists play an important role in detecting dental symptoms related to CeD, and this makes the dentist an integral part of the diagnostic team of CeD (Maloney et al., 2014 [10]; Mantegazza et al., 2016 [11]; Nieri et al., 2017 [12]; van Gils et al., 2017 [9]). When CeD is suspected, dental practitioners can liaise with the general medical practitioner to organize screening for CeD. Paul et al. [13] highlighted the dental manifestations of CeD to equip dental practitioners to aid the early diagnosis and initiation of treatment, especially for children with CeD. Karlin et al. [14] reviewed the dental manifestations of CeD to help pediatric dentists identify and refer atypically symptomatic patients to their pediatricians. It is now appreciated that age at diagnosis of CeD is rapidly changing due to the progression in the knowledge of the signs and symptoms of disease among doctors and patients. The average age of diagnosis of children in the most recent publications averaged around 8 years of age when the permanent teeth eruption has started. The apparently lower frequency of DEDs in adults may be caused by the fact that in many cases, the development of CeD might have taken place after the mineralization of dental crowns. Early recognition and diagnosis help in enabling the prompt implementation of a GFD, which results in better treatment and militates against dental complications. 

The aim of the present overview is to summarize the data of the literature from 2010 to March 2023 about dental manifestations of CeD and the role of dentists in identifying people who may have unrecognized CeD. Relevant studies before 2012 have been summarized by Krzywicka et al. [7].

## 2. Materials and Methods

Table 1 shows the PICOT methodology used for the present literature search. A comprehensive review of articles selected from MEDLINE, EMBASE and Google Scholar was performed by two independent operators [search protocol on Figshare license n.CC BY 4.0]. Additional studies hand-searched and found in the principal dental and gastroenterology journals were included for articles published in English from 2010 to 2023. The searched keywords were “c(o)eliac disease“ in combination with ”oral manifestations“, “oral health”, and “dental enamel defects”. We retrieved a total of 110 publications, of which 43 were reviews, expert opinions, or comments. We evaluated 47 original studies on the topic and reported the data of all of them. Additional papers were selected from personal files on CeD and by cross-referencing from the retrieved articles. Articles without abstract, such as case reports, commentaries, conference papers, and letters were excluded. 

## 3. Delayed Dental Eruption and Maturity

Several studies have shown that children with CeD tend to have more delayed dental eruption and maturation compared to healthy controls. This is attributable to the frequent delay in general growth and development found in pediatric CeD patients [15]. The poor nutritional status sometimes observed in CeD patients may play a causative role. Former reports on delayed dental eruption in relation to CeD were scant and controversial [7], and recent studies needed to generate clearness. In 2014, 30 Turkish children with biopsy-proven CeD and 30 without CeD, aged between 6 and 16 years, were enrolled at different hospitals to examine dental eruption [16]. Delayed dental eruption was observed in 10 CeD children (33%) and none of the controls. The difference was statistically significant (*p* < 0.05). To investigate dental eruption in potential and ascertained CeD children in comparison to healthy controls, a cross-sectional Italian study was performed including 50 ascertained CeD children, 21 potential CeD patients and 54 controls [17]. Clinical dental delayed eruption was observed in 19 ascertained CeD patients (38%) with an average value of 1.4 years of delay and in 9 potential CeD patients (42.8%) with an average of 1.7 years of delay. Among the control group, six healthy subjects (11.1%) presented a delay of tooth eruption. In contrast, an oral examination of 28 French children with CeD and 59 control children, all <12 year old with deciduous or mixed dentition, revealed that neither the CeD children nor the controls had delayed eruption [18]. Future investigations are urgently needed to confirm or disprove CeD and delayed dental eruption associations.

Similarly, there has been a limited number of studies on children with CeD in relation to delayed dental maturation and age. A clinical statistic Italian study on oral manifestations of CeD included 300 CeD patients, aged between 4 and 13 years (mean 8.16 years), and 300 healthy subjects, age-matched with a mean age of 8.29 years [19]. Regarding the prevalence of delayed dental maturation, the differences, observed between the CeD and control groups (20% vs. 8%), were highly significant (*p* = 0.0001). The same research group recruited 120 female patients (age range 12.0–12.9 years) to assess cervical vertebral maturation and dental age [20]. Among them, 60 subjects (group 1) were affected by CeD, while the control group (group 2) consisted of 60 healthy subjects, sex and age matched. The group 1 was subdivided, according to the period of CeD diagnosis, in group A (early diagnosis) and group B (late diagnosis). The assessment of skeletal-dental age revealed statistically significant differences between groups 1/2 and groups A/B (*p* < 0.001 each). Through the data analysis, it was possible to assess that the percentage of subjects with skeletal and dental age delay corresponds to 20% in healthy subjects, 57% in CeD subjects, 23% in CeD subjects with early diagnosis and 90% in CeD subjects with late diagnosis. To evaluate the presence of a possible relationship between the estimated delay in skeletal development and that in dental age, 70 Italian children affected by CeD (aged between 5.3 and 13.8 years) were selected [21]. The results showed how the dental age, which was clearly delayed in children affected by CeD, may be considered a reliable indicator of somatic growth and biological age. Furthermore, GFD had considerable beneficial effects on skeletal development in relation to the dental age. The delay of dental development decreases progressively from the time of diagnosis of CeD to the introduction of a GFD.

A Saudi Arabian case–control study examined delayed dental maturity in children with CeD compared to healthy controls to look for possible predictors of this manifestation [22]. Altogether, 104 children with biopsy-proven CeD and 104 healthy children (mean age of both groups 10.7 years) were recruited to compare delayed dental maturation based on difference between dental age and chronological age measured according to Demirjian’s method. CeD patients had a higher prevalence of delayed dental maturation than controls (63% vs. 3%) and also had a greater degree of delay than controls (7.9 vs. 7.0 months; *p* < 0.001). These findings were broadly in line with a number of previously published case–control studies, where a range of 20–70% for children with CeD vs. 7–20% for healthy controls were reported.

Altogether, most recent studies confirmed the relationship between delayed dental eruption and maturation with CeD. Most authors related these conditions to poor nutritional status, often observed in patients with active CeD. Because malnutrition can have irreversible effects on tooth development, early diagnostics toward CeD and the introduction of a GFD may be required. Therefore, pediatric dentists should consider the possibility of CeD in children, presenting delayed dental eruption and maturation, and their referral to pediatricians, so a screening for CeD can be undertaken. Further comprehensive investigations can add to our understanding of these dental complaints.

## 4. Dental Enamel Defects

**Figure 1:**
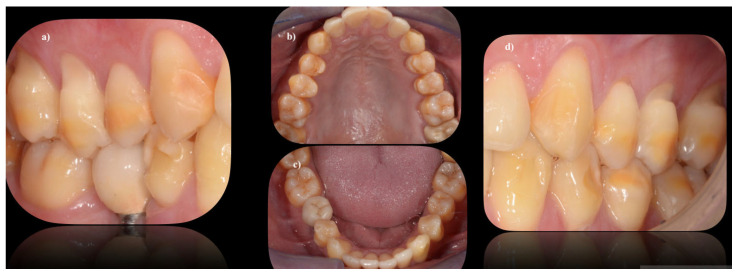
Evident structural enamel defects in a 40-year-old male CeD patient; (**a**) buccal surfaces of the right upper and lower canine, premolar and molar; (**b**) upper dental arch in occlusal view; (**c**) lower dental arch in occlusal view; (**d**) buccal surfaces of the left upper and lower canine, premolar and molar.

**Figure 2:**
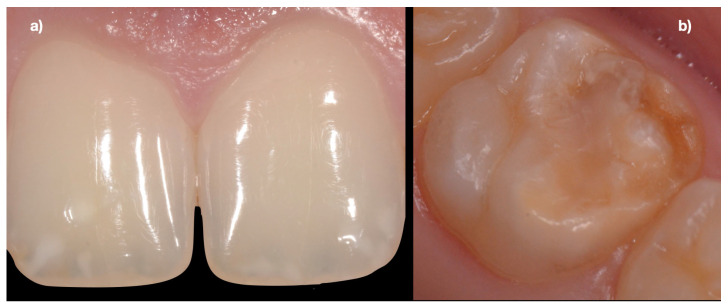
(**a**) Hypoplasia and opacity of the enamel with clearly defined margins on the maxillary central incisors in a 25-year-old female CeD patient; (**b**) Slight structural enamel defects of the occlusal surface, with an evident structural defect on the mesio-buccal cusp of the first maxillary right molar in a 17-year-old female CeD patient.

DEDs are the most frequently described dental manifestations in CeD and may be indicators of CeD, even when no other symptom of CeD is present (Figure 1 and Figure 2). DEDs associated with CeD have been the subject of many studies focusing on children. Krzywicka et al. (2014) have presented the corresponding investigations up to 2013 [7]. A systematic review and meta-analysis by Nieri et al. showed that CeD patients had a greater frequency of DED than healthy controls [12]. The following includes investigations on the relations between DED and CeD from 2012 to 2022. DEDs may occur in numerous systemic diseases, but the changes in CeD are highly specific. They appear symmetrically and chronologically in the same anatomical groups of teeth in all four quadrants of the dentition. A four-grade classification, proposed for the purpose of the assessment of DEDs [23], is shown in Table 2. A meta-analysis by Souto-Souza et al., including 45 studies and 2840 patients, revealed that DEDs, diagnosed using Aine’s method, were strictly related to CeD [24].

### 4.1. Frequency of DEDs in CeD

The frequencies of DEDs in children, adolescents, and adults with CeD compared to healthy individuals, presented in international studies from 2012 to 2022, are summarized in Table 3. 

#### 4.1.1. Children and Adolescents

Since 2012, a number of Turkish studies have aimed to investigate the prevalence of DEDs in young patients with CeD compared to healthy controls. A total of 35 patients diagnosed with CeD, aged 6 to 19 years, and 35 healthy individuals of the same age range participated in the study by Acar et al. [25]. DEDs were observed in 14 patients with CeD (40%). Of these 14 cases, 12 had defects of Grade I and 2 patients of Grade II. None of the subjects in the healthy group had DEDs (*p* = 0.05). In another study, 81 children with CeD (mean age 8.7 years) and 20 age- and sex-matched healthy children were examined for DED frequency [26]. Results revealed that DEDs occurred in 43 CeD patients (53%) and 5 control subjects (25%; *p* = 0.025). The presence of DEDs in 25 CeD patients, aged 4 to 16 years, and 25 age- and gender-matched healthy controls were studied by Cantekin et al. [28]. DEDs were observed in 12 out of 25 children in the CeD group (48%) and in 4 out of 25 control children (16%) (*p* = 0.01). In another Turkish study, 30 children with biopsy-proven CeD and 30 without CeD, aged between 6 and 16 years, were enrolled [16]. Twenty CeD patients (66.7%) had DEDs, none in the control subjects. Grade I was found in 14 subjects (46.6%) and Grade II in 6 subjects (20%); Grades III and IV were not observed. The most recent Turkish study showed that DEDs were present in 38,3 % of the children with CeD. The most recent diagnosis showed a lesser prevalence and intensity of enamel defects than those with earlier diagnosis [39].

The clinical evaluation of DEDs in Italian children included 50 subjects with ascertained CeD, 21 subjects with potential CeD, and 54 controls [17]. The mean age was 7.5, 6.9, and 8.8 years, respectively. The presence of specific DEDs was detected in 24 of 50 ascertained CeD patients (48%) and 4 of 21 potential CeD subjects (19%), with a statistically significant difference (*p* = 0.0328). Specific DEDs were completely absent in the healthy controls (0 of 54 subjects). In a recent Italian study, 114 pediatric patients (aged between 6 and 14 years) were divided into 3 groups with 38 participants each: CeD patients (CeD group), patients with malabsorption without CeD (non-CeD group), and healthy controls [37]. The CeD group showed more severe DEDs (68%) compared to the non-CeD group (39%) and the control group (29%). In the CeD group, 34% of patients were assigned to Grade I, 24% to Grade II, and 10% to Grade III defects. In the non-CeD group, 32% had Grade I and 8% Grade II defects, while no Grade II and III defects were observed in the control group. The evaluation of oral manifestations of French children, including 28 CeD patients and 59 controls (all aged < 12 years), revealed that CeD children had significantly more DEDs than the control group (68% vs. 34%; *p* = 0.004) [18]. In a study from Greece, 45 children with CeD (mean age 10.3 years) and 45 age-and gender-matched healthy children were examined for DEDs [33]. According to the clinical examination, specific defects were detected in 23 subjects of the CeD group (51.1%) and 5 subjects of the control group (11.1%) (*p* = 0.001).

A case–control study from Saudi Arabia, including 104 children with CeD (mean age 10.7 years) and 104 healthy children (mean age 10.7 years), revealed that CeD children had more DEDs than controls (70.2% vs. 34.6%; *p* < 0.001) [36]. Children with CeD were more likely to have Grade I (49.0%), Grade II (17.3%), and Grade >II defects (3.8%) than controls (22.1%, 10.6%, and 1.9%, respectively). Among a Brazilian cohort of 52 children with CeD, aged 2 to 15 years, 57.7% of the cases had specific DEDs, whereas, in the 52 age- and gender-matched controls, only 13.5% had specific DEDs (*p* = 0.00001) [29]. In a study by Cruz et al., 40 Brazilian children with CeD (mean age 11.2 years) and 40 age-matched non-CeD controls were selected for DED examination [32]. In the CeD group, 15 out of 40 subjects (37.5%) had signs of DEDs compared to 7 out of 40 subjects (17.5%) in the control group. Grade I defects were the most frequent in both groups, affecting 20 individuals: 13 among the CeD patients (32.5%) and 7 among the control individuals (17.5%).

All in all, the well-known correlation between CeD and DEDs in children has been confirmed by recent investigations. There is a direct relationship between DED and gluten exposure time: the older the pediatric patient is at the time of CeD diagnosis, the greater the number of teeth involved [37]. Therefore, early suspicion of CeD in children with DED by the pediatric dentist may help prevent severe consequences. Because DEDs might be the only manifestation of CeD, screening for CeD is highly recommended among children with DEDs, especially in the presence of underweight and hypocalcemia.

#### 4.1.2. Adults

It is well-known that late gluten exclusion by maintaining a GFD does not influence DEDs, which has been shown by the following investigations of adult CeD patients on a GFD. Dental examination was performed in 54 Italian CeD patients (mean age at diagnosis 31 years); at the time of evaluation, they all were on a GFD [27]. DEDs were observed in 46 of 54 patients (85%). Grade I defects were seen in 18 patients (33%), Grade II in 16 (30%), Grade III in 8 (15%), and Grade IV in 4 (7%). The study by Amato et al. [31] included 49 Italian patients affected by CeD (mean age at test: 31.8 years, mean time on GFD: 8.73 years) and 51 healthy volunteers (age at test: 30.5 years). DEDs were reported in 7 patients (14.3%) and in 0 controls (*p* = 0.002). Four patients had Grade I and three had Grade II defects. To evaluate dental manifestations in Indian adult patients with CeD, 118 subjects with biopsy-proven CeD (38 at diagnosis and 82 after a GFD for at least one year) and 40 controls were recruited [35]. All in all, 66.9% of CeD patients at diagnosis and 69.4% of CeD patients on a GFD had DEDs in comparison to 20% of controls. The similar frequencies of DEDs in newly diagnosed and long-time GFD-treated CeD patients confirmed that the damage of dental enamels has already been established and it is irreversible. Figure 1 and Figure 2 show examples of DEDs in adults with CeD. Therefore, the early diagnosis and treatment of CeD in adults is important for preventing the development of DEDs.

### 4.2. Frequency of CeD in DEDs

Whereas the higher prevalence of DEDs in CeD patients has been well documented by a number of studies, the prevalence of CeD in individuals with DEDs is scarcely investigated. The frequency of CeD among Egypt children with DEDs was evaluated by comparing 140 patients with DEDs and 720 age- and sex-matched controls for serum IgA and IgG TGA levels, specific for CeD [40]. CeD was more diagnosed in patients with DEDs (17.9%) compared to controls (1.0%) (*p* < 0.0001). The majority of non-CeD patients showed Grade I defects. In contrast, CeD patients suffered from Grades I, II, and III defects in CeD, showing that the degree of enamel damage was significantly increased in CeD patients compared to non-CeD patients. Further studies are necessary to strengthen the conclusions of these findings.

### 4.3. Pathomechanism of DEDs in CeD

The exact mechanism by which CeD leads to DEDs is yet not precisely clarified. The role of nutritional, genetic, and immunological factors, disturbing the normal process of amelogenesis, is still the focus of corresponding research. 

#### 4.3.1. Nutritional Deficiencies

Deficiencies of various micronutrients frequently present in active CeD may be responsible for DED development. In particular, malabsorption of calcium (hypocalcemia), phosphate, and vitamin D may disturb the process of amelogenesis. Regarding calcium supply, comparative studies of 140 children with DEDs (25 CeD cases) and 720 age- and sex-matched controls (7 CeD cases) revealed that there were significantly lower mean serum calcium levels (7.9 vs. 9.6 mg/dL) among patients with CeD compared to non-CeD individuals (*p* = 0.0001) [40]. On the contrary, mean serum phosphorus was not statistically different between CeD and non-CeD patients (3.5 vs. 3.8 mg/dL). The examination of 52 children with CeD indicated a significantly lower calcium/phosphorus ratio (1.35 vs. 1.58) in the primary dental enamel compared to the 52 control children (*p* = 0.0136) [29].

#### 4.3.2. Immunological Factors

In addition to nutritional deficiencies, the period of interruption of amelogenesis has been considered involved in DEDs, and autoimmune reactions against amelogenins and ameloblastin that direct the mineralization of enamel may play an important role. Munoz et al. analyzed the reactivity of sera from patients with CeD against gliadin and enamel-derived peptides [41]. Immunoblot analysis revealed that the most prominent component in enamel matrix derivative, identified by an amelogenin-specific antibody, was recognized by sera from patients with CeD. Based on these results, Sonora et al. (2016) analyzed the ability of anti-gliadin IgG, produced during untreated CeD, to recognize enamel organ structures [42]. Strong staining of the enamel matrix and the layer of ameloblasts was observed with serum samples from patients with CeD. High IgG reactivity was found against gliadin peptides and enamel matrix protein extract. These results strongly suggest a pathological role for antibodies to gliadin in DEDs.

To investigate amelogenin-specific antibodies in children with untreated CeD, blood samples from patients with CeD (*n* = 75) and healthy controls (*n* = 24) were analyzed for IgA and IgG reactivities to amelogenin by ELISA [43]. Whereas children with CeD had statistically significantly higher serum levels of anti-amelogenin IgA, only those with the most severe CeD (Marsh 3C) had significantly higher anti-amelogenin IgG immune reactivity than the controls. Blood samples from 32 CeD children with the highest IgA anti-amelogenin reactivity were selected for detailed IgA anti-amelogenin epitope mapping using 31 overlapping 10–22 mer peptides in ELISA [44]. The dominating reactivity was directed to six peptides in a 75-amino-acid-long central segment (sequence positions 75–150) and two N-terminal peptides (positions 13–41) included in the tyrosine-rich amelogenin peptide fragment, which is important for self-assembly.

#### 4.3.3. Genetic Factors

The genetic predisposition of CeD includes the human leukocyte antigen (HLA) class II genes HLA-DQ2.5, -DQ2.2, and -DQ8. The absence of these genes is a reliable negative predictor in the diagnostic procedure and is used to rule out the existence of CD [45]. Considering that CeD is related to specific HLA-DQB1 haplotypes, Erriu et al. tested whether the presence of the HLA-DQB1*02 allele could be a hypothetical cause behind the development of oral manifestations [46]. For this study, the oral condition and the presence of the HLA-DQB1*02 allele of 98 Italian patients (medium age: 35.9 years; range: 7–77 years), all affected by CeD and all on a GFD for at least one year, were examined. The statistical analysis showed that the absence of the HLA-DQB1*02 allele was predisposed to oral manifestations such as DEDs. Based on these findings, the following work aimed to verify whether the same evidence can be confirmed in pediatric patients [47]. Overall, 44 CeD patients with a median age of 9.9 years were studied. According to the clinical evaluation, DEDs were diagnosed in 17 patients (38.6%). HLA-DQB1∗02 distribution showed similarities with the previous work on adults [46]. The percentage of patients carrying two copies of the alleles was 38.6% and 40.9% showed heterozygosis, while only 20.5% did not carry the allele. DED diagnosis showed it was related to the presence or absence of the allele expression (*p* = 0.042).

## 5. Molar Incisor Hypomineralization

Molar incisor hypomineralization (MIH) is a qualitative and quantitative defect of the enamel structure treated as a separate disease entity of an unknown etiology [7]. MIH affects the first molars and incisors in the permanent dentition. It is caused by the lack of mineralization of enamel during its maturation phase due to interruption to the function of ameloblasts. Because the relationship between MIH and CeD has been uncertain, the occurrence of MIH in 40 CeD patients from Brazil was compared with 40 healthy controls [48]. The median age of the participants was 16.5 years (5–34 years) for both groups. Of the 80 participants, 10 presented MIH with 8 of those having CeD. Thus, CeD patients had 4.75 times the chance of occurrence of MIH than the control group (*p* = 0.044). In all the 978 evaluated teeth, 22 had MIH: 20 teeth in individuals with CeD and 2 teeth in those without CeD. All CeD participants with MIH presented the classic form (gastrointestinal symptoms) of CeD. They showed 17 teeth (85.0%) with demarcated opacities, 2 teeth (10.0%) with post-eruptive collapses, and 1 tooth (5.0%) with atypical restoration. The control group presented only demarcated opacities. In conclusion, CeD increased the chance of MIH, and dentists can assist in the diagnosis of CeD, when MIH has been detected. Further investigations with an enlarged number of patients and controls are needed.

## 6. Dental Caries

Previous investigations on the association between dental caries and CeD have been quite controversial regarding the frequency of caries in CeD patients and the influence of diet and oral hygiene on caries occurrence [7]. Recent studies, described in the following, could not clarify these contradictions. The classic DMFT/dmft index is one of the most common methods for calibrating caries (DMFT, permanent dentition; dmft, deciduous dentition) and was used by most researchers.

The aim of Italian clinical statistic studies by Costacurta et al. was to observe dental caries and calculate the DMFT/dmft index in relation to CeD [19]. Altogether, 300 children with CeD, aged between 4 and 13 years, and 300 healthy age-matched children were enrolled. Regarding dental caries frequency, the results demonstrated that CeD patients had higher caries indexes than healthy subjects in permanent teeth (DMFT 2.97 vs. 1.74; *p* = 0.0001) and deciduous teeth (dmft 2.31 vs. 1.42; *p* = 0.021). A total of 25 CeD patients aged between 4 and 16 years and 25 age- and gender-matched healthy controls were examined for the DMFT/dmft scores to determine the presence and distribution of dental caries in Turkish children [28]. The mean dmft values for the CeD and control groups were 3.25 and 4.56, respectively. However, the difference was not statistically significant (*p* > 0.05). The mean DMFT values for the CeD and control groups were 3.75 and 1.83, respectively, and statistically different (*p* < 0.01). To identify any influence of gender on the relationship between dental caries and CeD, 237 Italian adolescents and adults with CeD, aged > 14 years, were asked to fill in a questionnaire [49]. Among them, 182 were females, and 55 were males. Regarding caries prevalence, 49 of 237 subjects suffered from caries. Significant differences were observed between females (18%) and males (12%), indicating almost twice the susceptibility of females to caries. Investigations by Kalvandi et al. (2021) aimed to evaluate the CeD-specific serology in Iranian subjects with caries [50]. All in all, 120 children aged 3 to 12 years, referred to the dental clinic with enamel caries, were assessed by measuring serum IgA-TGA levels. The positive CeD serology rate of the studied population was 14 (11.6%). 

Other investigations could not demonstrate significantly increased caries cases in pediatric CeD patients compared to healthy controls. A study from Israel prospectively investigated three groups: newly diagnosed CeD (A), CeD treated with GFD (B), and a non-CeD control group (C) [51]. Group A comprised 30 children aged 1.4–15.5 years, group B 30 children aged 2.5–18 years, and group C 30 children aged 1.25–15 years). The mean DMFT/dmft indices were 1.5 ± 2.2 (group A), 2.0 ± 2.6 (group B), and 3.4 ± 3.7 (group C), respectively. No significant difference (*p* = 0.43) was found among the three groups, although there was a tendency toward a higher DMFT/dmft index in the control group. The caries frequency of 166 Italian patients with CeD, between 2 and 17 years of age, was compared to that of a control group with similar socio-demographic features [52]. The results revealed that the prevalence of caries was the same in the two groups (45% vs. 45%). Studies from Brazil compared 40 children with CeD (median age 16.5 years) with 40 age-matched healthy controls [32]. The prevalence of untreated caries in CeD patients (30.0%) was indeed higher compared to controls (22.5%) but not statistically different (*p* = 0.446). Even a lower mean DMFT (2.11) was found among another Brazilian cohort of 52 children with CeD (aged 2–15 years) compared to 52 age-and gender-matched controls (3.90; *p* = 0.0024) [29]. The multivariate analysis demonstrated that CeD acted as a protective factor in the caries experience. This result was explained by the fact that these individuals maintained a rigid GFD that usually contained fewer cariogenic foods than a normal diet, such as oatmeal, flour, and bread.

Most recently, a study demonstrated that the higher numbers of dental caries in permanent teeth of children with celiac disease may be related to Marsh type 2 mucosal damage.

In conclusion, the relation between caries and CeD and the effect of a GFD has yet to be elucidated neither in the former nor in recent studies. Clinical investigations should be encouraged to clarify the association between caries and CeD by including sufficient CeD and non-CeD patients.

## 7. Dental Plaque

Dental plaque is a biofilm of microorganisms (mostly bacteria) that is usually found between the teeth, on the front of teeth and behind teeth. Bacterial plaque is one of the major causes for dental decay and gum disease. To assess the occurrence of dental plaque in Israeli pediatric individuals with CeD, 30 children with newly diagnosed CeD, 30 children with GFD-treated CeD, and 30 healthy children were enrolled [51]. There was a statistically significant difference in plaque debris index (*p* = 0.02) among the three groups. The highest plaque index mean value (1.88) was found in the newly diagnosed CeD group, followed by the controls (1.42), whereas the lowest mean value (1.31) was found in the GFD-treated CeD group. The latter finding could not be explained by salivary properties or bacteria but rather by better oral hygiene. Children receiving GFD brushed their teeth and used fluoride significantly more often than other children in the study. A recent study found that dental plaque debris index was similar in with CeD on a long-lasting gluten-free diet and those recently diagnosed [39]. Further research is necessary to confirm these findings.

## 8. Teeth Enamel Wear

**Figure 3:**
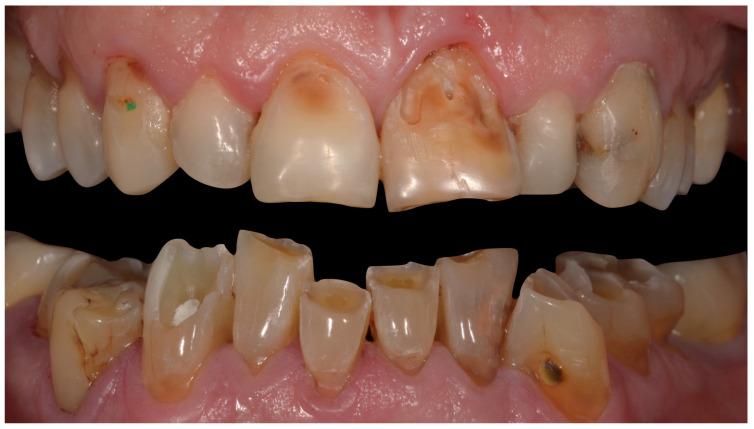
Wide enamel–dentine wears, associated with non-carious cervical lesions in a 60-year-old male CeD patient.

Teeth enamel wear refers to the loss of dental substance by means other than caries, as a result of a combination of attrition, abrasion, and erosion (Figure 3). Although tooth wear is frequent in the general population and increases with age, a study suggests that CeD adults shave increased dental wear likely due to bruxism when compared to non-CeD controls [31]. CeD and bruxism are associated with sleep disorders, nutrient deficiencies, and psychological problems. This correlation could explain the frequent occurrence of enamel wear in CeD patients.

## 9. Periodontitis

Periodontitis, also known as gum disease, is an inflammatory disease affecting the tissues surrounding the teeth. In its early stage, the gums become swollen and red and may bleed (gingival bleeding). Ultimately, the gums can pull away from the tooth, and the teeth may fall out if the disease is not diagnosed and treated in time. Periodontitis is due to bacteria in the mouth infecting the tissue around the teeth. To evaluate the factors that influence the needs for periodontal treatment, 35 Greece children and adolescents with CeD (aged 4–16 years) were examined using the simplified gingival index and the periodontal screening and recording index [53]. The results revealed that most of them needed treatment for gingivitis (60.0%) and a minor proportion of the subjects had a healthy periodontium (34.3%). The simplified gingival index correlated statistically significantly with the presence of a coexisting disease, the frequency of tooth brushing, bleeding upon brushing, and oral malodor. The periodontal status of the subjects with CeD did not have any specific characteristics but they had similarities to the status of the general pediatric population. A comparative examination of health was not performed.

To investigate whether signs of periodontitis are associated with CeD among US adults, the National Health and Nutrition Examination Survey (NHANES) 2009–2012 enrolled 6661 subjects with full-mouth periodontal examination and CeD-specific serological testing [54]. CeD was defined as self-reported physician diagnosis while on a GFD (diagnosed CeD) or positive serology (undiagnosed CeD). Multivariable linear and logistic models were used to regress the attachment loss (AL) or mean probing depth (PD) outcomes across CeD categories (no CeD, *n* = 6232; diagnosed CeD, *n* = 13; undiagnosed CeD, *n* = 33). The mean levels of % AL among individuals without CeD and with diagnosed or undiagnosed CeD (18%, 16%, and 15%, respectively) were not significantly different (*p* = 0.72). In contrast, the mean PD levels among those without CeD and with diagnosed or undiagnosed CeD (1.49 mm, 1.36 mm, and 1.31 mm, respectively) were significantly different (*p* = 0.03 for any difference). In conclusion, CeD was associated with modestly lower levels of mean probing depth but was not associated with mean attachment loss.

Nota et al. [49] demonstrated that the prevalence of gingivitis in CeD patients was clearly correlated with the age of the subjects. Among the group aged 15–25 years (*n* = 51), signs and symptoms of gingivitis could not be detected (0%). The prevalence increased dramatically (90%) in the group aged 26–55 (*n* = 136) and distinctly (71%) in the group aged > 55 years. Altogether, larger studies are necessary to enhance precision and strengthen conclusions and to estimate the impact of a GFD on periodontal indices.

## 10. Conclusions

Apart from gastrointestinal complaints, CeD may present a wide spectrum of extra-gastrointestinal symptoms. Several studies underlined that certain dental manifestations are well-recognized signs of CeD and in patients with yet undiagnosed CeD, these can sometimes be the only presenting features (Table 4). Moreover, visible dental manifestations of CeD in children likely cause a reduction in their quality of life [55]. The association between CeD with delayed dental maturation in children and with DEDs in children and adults have been confirmed in recent years. Accordingly, DEDs and the delay of dental maturation are effective risk indicators of CeD. For these reasons, dentists play a fundamental role in the early diagnosis of CeD and may help prevent its progress and long-term complications. When the diagnosis of CeD and its treatment with a GFD do not occur in time, the damage by DEDs remains irreversible. Therefore, patients with specific DEDs should be screened for CeD even in the absence of gastrointestinal symptoms.

Although recent studies supplemented older studies on the relationship between CeD and other dental complications, such as delayed dental eruption, MIH, caries, plaque, and periodontitis, clear conclusions could not be reached. Moreover, the role of a GFD in the improvement in symptoms remains to be solved. The results were, in parts, contradictory and not significant or representative, because either only a few studies were performed and small numbers of patients and controls were included, or the studies presented high heterogeneity criteria and methods for evaluations. Therefore, further comprehensive investigations are necessary to clarify the association between dental manifestations and CeD and the effects of a GFD by including a sufficient number of CeD and non-CeD patients.

## Figures and Tables

**Table 1:** PICOTS methodology used in the systematic search about oral and dental manifestations in celiac disease.

PICOTS Parameter	Inclusion Criteria	Exclusion Criteria
Patients	Patients from 0 to 99 years with celiac disease	Another intestinal disease not universally recognized as celiac disease
Intervention	Not applicable	
Comparator	Non-celiac individuals	
Outcome	The presence/absence of dental manifestations such as dental enamel defects, plaque, periodontitis and aphthous stomatitis	
Time	Studies published from 2010 to March 2023	
Study design	Connections between oral/dental pathology and celiac disease	Reviews, expert opinions, comments, letters to editors, case reports, conference reports, and studies not published in English.

**Table 2:** Grading of the CeD-related DEDs adapted from Aine (1986) [23].

*Grade I: Presence of defect in color of enamel* Single or multiple yellow or brown opacities with clearly defined or diffuse margins; a part or the entire surface of enamel is without glaze.
*Grade II: Slight structural defects* Enamel surface is rough, filled with horizontal grooves or shallow pits; light opacities and discoloration may be found; a part or the entire surface of enamel is without glaze.
*Grade III: Evident structural defects* A part or the entire surface of enamel is rough and filled with deep horizontal grooves which vary in width or have large vertical pits; large opacities of different colors or strong discoloration may be in combination.
*Grade IV: Severe structural defects* Shape of tooth changed: tips of cusps are sharp-pointed and/or incisal edges unevenly thinned and rough; the enamel thinning is easily detectable, and the lesions margins are well defined; lesions may be strongly discolored.

**Table 3:** Frequency [%] of dental enamel defects (CeD patients vs. controls).

Author (Year)	Country	Adults		Children/Adolescents	
		n ^a^	%	n ^a^	%
Acar (2012) [25]	Turkey	-	-	35 vs. 35	40 vs. 0%
Ertekin (2012) [26]	Turkey	-	-	81 vs. 20	53 vs. 25%
Trotta (2013) [27]	Italy	54 ^b^	85 ^b^	-	-
Bramanti (2014) [17]	Italy	-	-	50 vs. 54 ^c^	48 vs. 0% ^c^
Cantekin (2015) [28]	Turkey	-	-	25 vs. 25	48 vs. 18%
de Carvalho (2015) [29]	Brazil	-	-	52 vs. 52	58 vs. 14%
De Queiroz (2017) [30]	Brazil	-	-	45 ^b^	55.6%
Amato (2017) [31]	Italy	49 vs. 51	14 vs. 0%	-	-
Bicak (2018) [16]	Turkey	-	-	30 vs. 30	67 vs. 0%
Cruz (2018) [32]	Brazil	-	-	40 vs. 40	38 vs. 16%
Zoumpoulakis (2019) [33]	Greece	-	-	45 vs. 45	51 vs. 11%
Macho (2020) [34]	Portugal	-	-	80 vs. 80	55 vs. 27.5
Ahmed (2021) [35]	India	118 vs. 40	68 vs. 20%	-	-
Alsadat (2021) [36]	Saudi Arabia	-	-	104 vs. 104	70 vs. 35%
Villemur-Moreau (2021) [18]	France	-	-	28 vs. 59	68 vs. 34%
Ludovichetti (2022) [37]	Italy	-	-	38 vs. 38	68 vs. 29%
Elbek-Cubukcu (2023) [38]	Turkey	-	-	62 vs.64	11.7% vs. 0%as MIH
Bulut (2023) [39]	Turkey	-	-	78 ^b^	38.3%

^a^ Number of subjects. ^b^ Only CeD patients. ^c^ Specific DEDs; ascertained CeD vs. controls.

**Table 4:** Dental manifestation of celiac disease.

*Delayed tooth eruption:* children with celiac disease may experience delayed eruption of their permanent teeth, which can affect their bite and overall dental health. *Dental enamel defects:* celiac disease can cause enamel defects, which appear as white or brown spots, pits, or grooves on the teeth and can make the teeth more vulnerable to decay. *Caries:* individuals with celiac disease may be more prone to caries due to a combination of factors, including poor enamel quality, reduced saliva production, and an altered oral microbiome. *Plaque:* a single study shows that the biofilm covering the teeth of untreated celiac children seems to be different from controls and children on a gluten-free diet. *Enamel wears:* Celiac patients show more frequent dental enamel wear than controls. It is possible that celiac patients have more frequent bruxism than controls. *Periodontitis:* Celiac disease may also increase the risk of gum disease, a condition in which the gums become swollen, red, and tender. Gum disease can lead to tooth loss if left untreated.

## Data Availability

All data collected during this study are included in this paper.
